# The relationship between health and migration: exploring social capital and mobility preferences in China’s urbanization process

**DOI:** 10.3389/fpubh.2025.1575893

**Published:** 2025-05-22

**Authors:** Peng Ying Mei, Jialu You, Wei Wen Jun

**Affiliations:** ^1^Sociology Department, Guizhou Minzu University, Guiyang, Guizhou, China; ^2^Institution of Finance and Economics Department, Shanghai University of Finance and Economics, Shanghai, China; ^3^Business Department, Zhongnan University of Economics and Law, Wuhan, Hubei, China

**Keywords:** health status, migration, social networks, class mobility, China

## Abstract

**Purpose:**

Health status is a key determinant of life outcomes, including economic performance, educational attainment, and social integration. However, its impact on migration preferences remains underexplored. This study investigates whether health status significantly influences migration decisions in China, with a particular focus on the role of social capital and class mobility expectations.

**Methods:**

This study utilizes data from the China Labor Dynamics Survey (CLDS) collected between 2012 and 2018. Multiple regression analyses were conducted to assess the relationship between self-reported health, occupational quality, social capital, and migration decisions. The mediating role of social networks and the moderating effect of class mobility expectations were also examined.

**Results:**

The analysis reveals that individuals with better health are more likely to migrate, especially in pursuit of improved employment opportunities in urban areas. Social networks mediate the relationship between health and migration by providing resources and support that facilitate mobility. Furthermore, class mobility expectations moderate this relationship: healthier individuals with positive prospects tend to stay in their current location, while those who have experienced upward mobility are more likely to migrate.

**Conclusion:**

This study highlights the significant role of health status in migration decisions and emphasizes the importance of social networks and class mobility expectations. Policies aimed at improving health and strengthening social support systems could enhance social equity and mobility, particularly in developing countries.

## Background

Health issues are increasingly recognized as being intertwined with social, political, economic, and cultural contexts globally, complicating comprehensive and empirical examinations of this subject. Research on health issues often intersects with global scientific advancements and economic development, particularly in the realm of human relationships (1). At the health level, numerous studies have highlighted factors such as healthcare inequality, exacerbated by economic vulnerabilities, especially during the COVID-19 pandemic. These studies demonstrate that negative health outcomes are prevalent among migrants, influencing their preferences and behavior (2). This underscores the critical relevance of health to the economic welfare and behavioral patterns of immigrants worldwide.

**Figure 1:**
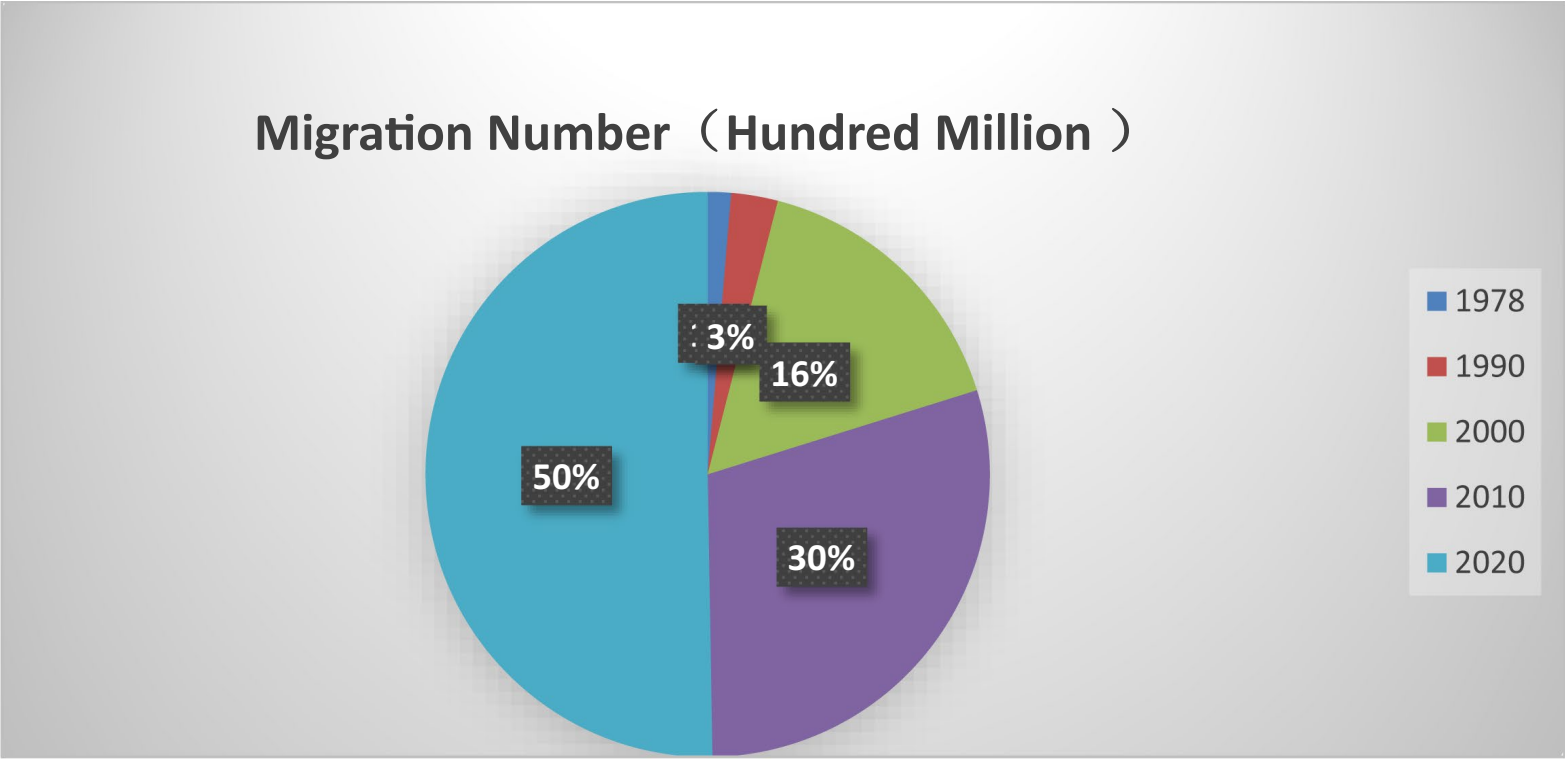
China migration number (1978–2020).

In China, the world’s most populous country according to the 2020 Chinese census, the immigrant^1^ population has reached one million, accounting for approximately 18% of the total population. The country has witnessed a dramatic rise in immigration, from 128 million in 1978 to 830 million in 2019, spurring rapid urbanization and economic growth (Figure 1). Despite this, high savings rates continue to pose challenges to China’s economic development, particularly in advancing its circular economy (37). Data indicates that consumption rates post-immigration increased by 10%, contributing to a GDP rise to five thousand dollars. Thus, immigration is emerging as a new driver of economic growth in China (40).

**Figure 2:**
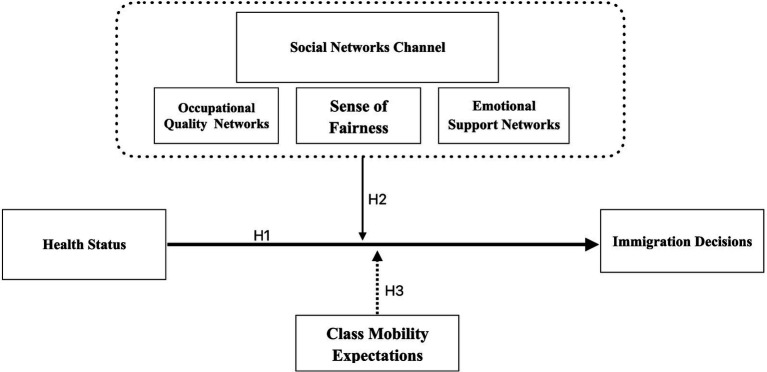
Harris-Todaro (HT) framework.

Health’s role in migration decisions has been largely overlooked in migration studies, which traditionally focus on economic and geographic factors. Recent studies have suggested that self-rated health significantly influences migration preferences. Healthier individuals tend to migrate more, as they are better able to handle the physical and emotional challenges of relocation. While much research has concentrated on economic factors like income and housing, health remains underexplored, particularly in rapidly urbanizing countries like China (Figure 2).

Amoah (3) and Kotschy (31) found that health is linked to longer life expectancy, lower mortality, and better health awareness, all of which influence migration decisions. Zhao (4) and Wang (35) also found that health disparities tied to income inequality affect migration preferences, with individuals in better health more likely to migrate. These studies show health plays a key role in migration, but how it impacts migration decisions through mechanisms like social networks and class mobility expectations remains underexplored.

Social networks are essential in migration. They provide resources, support, and information, all of which can ease the migration process. Luo (5) and Zhang et al. (36) showed that strong social networks improve health outcomes and help migrants adapt to new environments. Tutu et al. (6) found that social networks provide migrants with access to healthcare and other resources. Additionally, Aartsen et al. (7) and Liu et al. (33) highlighted how individuals with higher socioeconomic status (SES) have stronger social networks, which reduce loneliness and improve health, making migration more likely. However, the link between self-rated health and social networks in shaping migration preferences is still not well understood.

Class mobility expectations also affect migration. Individuals who believe they can improve their social status without moving are less likely to migrate. Wu and Chang (8) showed that those who have already experienced upward mobility or believe migration will improve their social standing are more likely to migrate. These findings suggest that social mobility expectations influence migration, but more research is needed to explore how these expectations interact with health.

Finally, health shapes perceptions of fairness and social justice, which also affect migration decisions. Bernard et al. (9) found that individuals with mobility impairments report lower life satisfaction and more negative emotions, largely due to their experiences with social injustice. These perceptions of unfairness may motivate individuals to migrate, but the role of health in shaping these perceptions is still not fully explored.

While health is an important factor in migration decisions, few studies have examined how self-rated health interacts with social networks and class mobility expectations to influence migration preferences. This study fills this gap by examining how these factors work together to shape migration decisions. By focusing on microeconomic factors like health, this study provides a more comprehensive understanding of the health-migration relationship.

This study aims to fill this gap by exploring how individual and family health status influences migration preferences. By focusing on microeconomic factors, such as self-rated health and its impact on mobility, this study provides a new perspective that integrates health as a key factor in migration decisions, alongside traditional factors like income and education.

This study uses the Harris-Todaro (HT) migration model to analyze how health influences migration preferences. While the HT model typically focuses on economic factors like wage differences between regions, this study extends the model by adding health as an important factor in migration decisions. Health capital, as part of human capital, affects the costs and benefits of migration. People in better health are more likely to migrate, as they are better equipped to deal with the challenges of relocation and can potentially be more productive in their destination regions (10).

This study contributes in three ways: first, it provides a microeconomic analysis of how health influences migration preferences, using individual-level data; second, it extends the Harris-Todaro model by incorporating health as a central factor in migration decisions, offering a more complete understanding of migration dynamics; third, it explores the mechanisms through which health influences migration, such as social capital, occupational quality, and class mobility expectations. This provides a deeper understanding of how health interacts with other factors to influence migration preferences.

In conclusion, this study aims to provide a more comprehensive understanding of how health status influences migration preferences, particularly in the context of China’s ongoing urbanization. By including health in migration decision-making, this study contributes new insights into the factors that shape migration preferences, particularly in developing countries experiencing rapid economic changes.

### The Harris Todaro theory framework for understanding migration preference

#### Harris-Todaro theory framework

The HT economic framework posits that significant developmental gaps exist between countries or regions, and migration serves as a form of human capital investment that allows individuals to secure better income, educational opportunities, and living standards (11, 39). A fundamental assumption of the HT framework is that migration is homogeneous and costless. However, subsequent studies have demonstrated that individual characteristics—such as education level, family social capital, social networks, gender, and marital status—significantly influence migration decisions (12, 13).

The Harris-Todaro (HT) economic framework posits that migration is a response to significant developmental disparities between regions or countries. It suggests that migration functions as an investment in human capital, enabling individuals to access better economic opportunities, higher educational standards, and improved living conditions (11, 39). A foundational assumption of this model is that migration is homogeneous and costless. However, more recent studies indicate that migration decisions are profoundly influenced by individual characteristics, such as education, family social capital, social networks, gender, and marital status (12, 13).

Health capital, as an essential component of human capital, plays a significant role in shaping migration decisions. Health status affects migration outcomes by influencing labor supply and investment in education. Good health enables individuals to work longer hours, earn higher incomes, and invest more in education, thereby enhancing their bargaining power in the labor market (10, 14). From a life-cycle perspective, better health contributes to higher lifetime income, which allows individuals to sustain stable consumption levels and leisure time without extending working hours due to longevity. Moreover, healthier individuals are more likely to remain in high-productivity conditions, benefiting from higher education and reduced labor intensity.

The existence of a “health migration effect” has been confirmed in various studies examining international migration, including China’s internal population movements (15). However, the mechanisms by which self-rated health levels influence migration decisions are still not well understood, and the role of health self-selection in migration is underexplored. As noted by Sjaastad (16) and Iranzo and Peri (17), migration involves both material and psychological costs, and individuals will choose to migrate only when the benefits outweigh these costs. Therefore, the relationship between health and migration preferences warrants further investigation, particularly in terms of health assessments and how these affect migration decisions.

### The preference function of immigrants

Building on the analysis of the social network mechanism and the Harris-Todaro (HT) economic framework, we propose a preference function to investigate the relationship between health status and immigrants’ preferences. The individual utility function (*μ*) is influenced by career prospects (*η*) and social mobility expectations (*φ*), Migration costs are comprised of both material and psychological costs, represented as $c_{\mathrm{pij}}^{t}$ and $c_{\mathrm{mij}}^{t}$ respectively. The material costs include living, education, and identification policy costs^2^ (40), while psychological costs include mood disorders and anxiety stemming from migration preferences (44).

we assume that the labor market is perfectly competitive, and both utility functions and cost of migration are influenced by the individual’s social network and the perception of society. Migration costs are determined by regional characteristics, individual attributes, social network size, and perceptions of fairness, represented as $c=c_{\mathrm{ij}}^{t}(\tau ,\partial ,\delta ,\lambda )$. Referring to Sjaastad (16), this study assumes that an individual’s utility at the original residence is presented as $u_{j}^{t}$, the utility at the destination is denoted by $u_{i}^{t}$, expected cost of migration is represented as $c_{\mathrm{ij}}^{t}$, the net benefit of migration from place i to place j is represented as:


1 $$u_{j}^{t}(\tau_{j},\varphi ,\eta_{j},u_{j})-u_{i}^{t}(\tau_{i},\partial ,\eta_{i},\mu_{i})>c_{\mathrm{ij}}^{t}(\tau ,\partial ,\delta ,\lambda ))$$

Equation 1 indicates that migration will occur if the net benefit exceeds the costs. In this study, the migration utility function is further refined to incorporate both self-assessments of health and external health assessments, which are essential in determining migration preferences:


2 $$\begin{array}{l} \;P_{V_{j}}=\sum_{t=1}^{T}\frac{\left(u_{j}^{t}-u_{i}^{t}-c_{\mathrm{ij}}^{t}\right)}{{\left(1+r\right)}^{t}}\\ =\sum_{t=1}^{T}\frac{\left(u_{j}^{t}(\tau_{j},\hat{\partial },\eta_{j},\mu_{j})-u_{i}^{t}(\tau_{i},\hat{\partial },\eta_{i},\mu_{i})-c_{\mathrm{ij}}^{t}(\tau ,\partial ,\delta ,\lambda )\right)}{{\left(1+r\right)}^{t}} \end{array}$$

The Equation 2 means that the higher the net benefit from the migration decision, the more individuals prefer to migrate.

### The current research

This study explores the role of self-rated health in migration decisions using the Harris-Todaro (HT) economic framework, with a focus on the mediating role of social networks and the moderating role of class mobility expectations. While existing literature has explored the influence of social networks on health outcomes Li (32) and migration preferences Kotschy (31), few studies have examined how self-rated health interacts with these networks to shape migration choices.

Social networks are crucial resources that impact both the frequency of health information searches and overall health outcomes. Studies show that social networks play an important role in improving individuals’ health by providing information, emotional support, and access to healthcare resources (5, 6, 29). For example, Zhang (41) found that social networks significantly enhance the physical health and quality of life of older adults, with neighborhood networks having a stronger impact on healthy aging than family or friend networks. Similarly, Daniela (18) and Song (34) highlighted that individual with higher socioeconomic status (SES) benefit more from their social networks, while marital relationships and social participation help reduce health inequalities, particularly for those with lower SES. Aartsen et al. (7) further explored how stronger social networks among individuals with higher SES help combat loneliness, leading to better health outcomes.

Building on these findings, this study also examines how health levels and perceptions of fairness affect migration decisions. Health inequalities are a persistent global issue, and physical health has a substantial impact on mental well-being, especially concerning social justice perceptions. For example, Bernard et al. (9) and Chen (28) found that individuals with mobility impairments experience more negative emotions and lower life satisfaction compared to their healthier counterparts, primarily due to feelings of social exclusion and inequality.

To enhance our understanding of these dynamics, this study uses the Harris-Todaro economic framework to develop a utility preference function that connects health and migration preferences through social network mechanisms. Specifically, we aim to test whether health significantly influences migration decisions (Hypothesis 1), the mediating role of social networks in this relationship (Hypothesis 2), and the moderating role of class mobility expectations (Hypothesis 3). These hypotheses will help clarify how health impacts migration preferences and how social networks and perceptions of fairness influence this relationship.

## Methods

### Data collection and participants

This study uses data from the 2012–2018 China Labor Force Dynamics Survey (CLDS). The CLDS was chosen because it has a strong sampling methodology and covers a wide range of topics. The survey uses a multi-stage, multi-level probability sampling method, which ensures a representative sample of China’s diverse population. It is the first survey in China to use a rotating sample tracking method, which helps capture changes in the socio-economic environment while keeping the strengths of cross-sectional surveys.

The sample is divided into four groups, with each group followed for four consecutive rounds over 6 years. After 6 years, the group is withdrawn, and a new rotating sample is introduced to maintain continuity. This design helps ensure that the data remains representative and reliable, especially in tracking health characteristics among the mobile population.

The CLDS targets the working-age population aged 15–64 and includes data on education, employment, labor rights, occupational mobility, health, job satisfaction, and overall well-being. The survey includes 401 villages, 14,226 households, and 21,086 individuals, covering 29 provinces and cities across China.

This study uses the CLDS dataset, which includes longitudinal data ideal for studying migration decisions. Probit and Logit regression models are used to analyze binary outcomes, such as whether a person migrates or not. These models are useful because they estimate the probability of migration based on health status and other factors, while controlling for potential confounding variables.

However, Probit and Logit models have some limitations. First, both models assume that the error terms are independent and identically distributed, which may not be true if there is unobserved heterogeneity. Second, migration decisions could be influenced by factors not included in the model, leading to potential endogeneity. These issues could result in biased estimates if not addressed. To reduce bias, propensity score matching (PSM) is used to control for observable confounding factors. This method helps ensure that migrants and non-migrants are similar in terms of the characteristics we observe, which reduces bias.

Additionally, instrumental variables (IV) are used to deal with potential endogeneity caused by unobserved factors. IVs are chosen based on their relevance to migration but not directly affecting health status, allowing us to isolate the external factors that influence migration decisions.

In summary, this study uses the CLDS dataset and Probit and Logit regression models to examine how health status affects migration decisions. While these models are useful for binary outcomes, their limitations are addressed through propensity score matching and instrumental variables, making the results more reliable. This approach provides a thorough analysis of how health influences migration preferences while accounting for potential biases and endogeneity.

## Measures

### Primary explanatory variable: migration status

Migration status was determined by the question: “Have you been away from your household location for more than 6 months?” Respondents who answered “yes” were classified as migrants and assigned a value of “1,” while those who answered “no” were classified as non-migrants and assigned a value of “0.”

### Health status: self-rated and objective assessments

Health status was a key variable in this study, assessed through both self-rated health and objective assessments by others. To reduce self-selection bias, two questions were used from the CLDS survey: “How do you rate your current health?” for self-assessment, and “How would you rate the respondent’s health?” for external assessment. Responses were recorded on a scale from “very healthy” to “very unhealthy,” which was then converted to a numeric scale from 5 (very healthy) to 1 (very unhealthy), where higher scores indicated better perceived health.

### Control variables

Several control variables were included to account for factors that might influence migration preferences. These variables included individual characteristics such as age, gender, education level, and parents’ education level, as well as regional characteristics like party membership status and province of residence (19).

The basic statistics for the research sample show an average self-rated health level of 3.583, with a range from 1 to 5, indicating variation in health status among respondents. The average age of participants is 42.6 years, ranging from 16 to 59, with the squared age variable averaging 2021.302. Approximately 7.9% of respondents are members of the Communist Party of China (CPC), suggesting relatively low political affiliation. The average education level among respondents is 2.727 years, with a maximum of 23 years, though some reported no formal education. The average education level of respondents’ parents is 2.701 years, indicating generational differences in educational attainment. Job satisfaction had an average score of 2.996, showing that respondents generally feel moderately satisfied with their current jobs. The reported income ranges from 2.97 to 11.38, with an average of 7.987, reflecting a broad income distribution in the sample. These characteristics offer a detailed snapshot of a diverse population, providing valuable context for understanding health status and migration preferences.

### Distributional analysis

**Figure 3:**
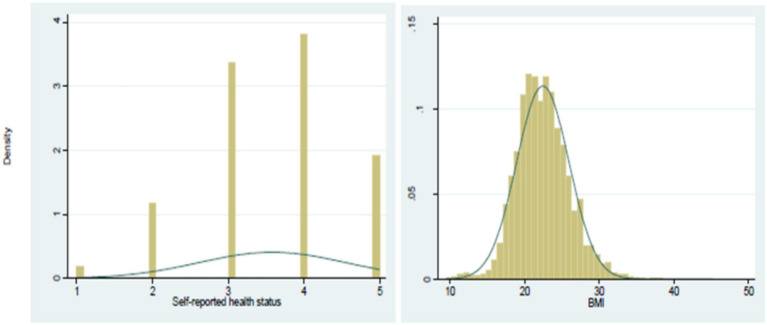
Distribution of self-reported health status and BMI in the estimation sample.

Figure 3 shows the distribution of key variables across the total sample, migrant sample, and non-migrant sample. The data indicates that the self-rated health of the non-migrant population (mean = 3.557) is lower than both the migrant population (mean = 3.818) and the total sample (mean = 3.583). This suggests that migrants generally have better health, supporting the “health migration hypothesis,” which posits that individuals in better health are more likely to migrate from rural to urban areas.

Further analysis reveals that the average age of migrants is around 35 years, significantly younger than the total sample average. This age group is often at a key stage in career development, balancing professional growth with personal life, and seeking career opportunities that align with long-term goals.

Additionally, migrants tend to have higher levels of education, better parental education, and are more likely to be CPC members, suggesting that individuals with more resources and higher social status are more mobile. Migrants also report higher levels of job satisfaction and income, indicating smoother career progression.

## Results

### The effect of health on immigrant mobility

This study explores the impact of health status on migration preferences, particularly focusing on the tendency of individuals to migrate from rural to urban areas. The underlying hypothesis is that healthier individuals face lower psychological and material costs associated with migration, thereby making them more likely to relocate.

To test this hypothesis, we employed a multiple regression analysis, controlling for a range of individual characteristics (such as age, gender, and education level), household factors, and province-level fixed effects. The regression model is presented as follows:


(3) $$\;{\mathrm{imm}}_{i}=\partial +\beta_{1i}\;\text{health}+\beta_{2i}X+\mu$$

In Equation 3, the X represents control variables; theμ defied as measuring error. And the dependent variable is a measure of whether the individual has been mobile, i.e., whether the respondent has left the household registration area for more than 6 months, with a value of “1” for mobile and “0” for non-mobile; it indicates the respondent’s self-rated health level, with a value of “1” for “very unhealthy” and “5” for “very healthy”; it indicates the control variables, i.e., the respondent’s age, gender, education level, whether the respondent is a member of the Chinese.

Table 1 analyzes the relationship between health status and the propensity to migrate using data from the CLDS (2012–2018). In different models such as OLS, Probit, Logit and Multilevel Logit, the findings consistently show a significant positive relationship between health status and the likelihood of migration.

**Table 1:** The regression of health status on migration preference.

Variables	OLS (1)	OLS (2)	Probit (3)	Probit (4)	Logit (5)	Logit (6)	Multiple level logit (7)
Health	0.0254***	0.0178***	0.149***	0.0359**	0.288***	0.0644**	
	(10.83)	(7.00)	(10.57)	(2.99)	(10.55)	(2.08)	
Individual health							0.0162***
							(3.52)
Household health							0.0212***
							(4.21)
City health							0.0042***
							(2.82)
Province health							0.0021**
							(2.14)
Politic		−0.0198*		−0.121		−0.274*	−0.0198*
		(−2.21)		(−1.82)		(−2.15)	(−2.21)
Age		0.00415***		0.0523***		0.130***	0.0049∗∗∗
		(4.59)		(3.51)		(4.22)	(4.59)
age2		−0.001*** (−3.51)		−0.093*** (−3.21)		−0.0223*** (−3.10)	−0.001*** (−3.51)
edu		0.0116 (1.12)		0.0279 (1.57)		−0.0661 (−1.81)	0.0116 (1.10)
parents_edu		−0.00415*		−0.0253*		−0.0440*	−0.0253∗
		(−1.96)		(−2.10)		(−2.15)	(−2.11)
Province_FE	No	Yes	No	Yes	No	Yes	Yes
Time_FE	No	Yes	No	Yes	No	Yes	Yes
_cons	0.0113	0.170***	−1.814***	−1.329***	−3.236***	−2.747***	2.8521***
*N*	16,244	11,635	16,244	11,635	16,244	11,635	16,244
*R* ^2^	0.217	0.191	0.217	0.211	0.211	0.191	0.2118

*t* statistics in parentheses. *p < 0.10, **p < 0.05, ***p < 0.01.

In the OLS model, in the first specification, each unit increase in health status increases the likelihood of migration by 2.54%. When additional control variables are added, this effect is still significant, but slightly lower, at 1.78 percent. Probit and logit models support these findings: in the probit model, an improvement in health initially increases the probability of migration by 14.9 percent, and by 3.59 percent after controlling for other factors. Similarly, the logit model showed that improved health increased the probability of migration by 28.8%, decreasing to 6.44% after controlling for other factors.

Multiple logistic regression analyses examined the effect of health status on migration preferences at different levels (individual, household, city and province). Health status was measured at each level to understand its full impact on migration preferences. Individual health, usually assessed through self-reported health status or physical health assessments, reflects an individual’s ability to migrate. Family health considers the overall health status of household members, including chronic diseases or care needs, which can influence migration decisions. Urban health is measured through public health indicators such as disease prevalence or access to health care, reflecting the broader context that may encourage or discourage migration. Provincial health status is assessed through regional health statistics, such as average life expectancy and health-care infrastructure, which may influence migration decisions on a broader scale.

Separating these dimensions in a regression analysis is an attempt to understand how each aspect of health affects migrant behavior. This approach also helps ensure the reliability of the findings and provides a clearer understanding of the factors that drive migration.

The results show that health status at the individual and family levels has a significant positive effect on the likelihood of migrating, with t-values of 3.52 and 4.21, respectively, suggesting that individuals with better individual and family health status are more likely to migrate. Health status at the city and provincial levels also positively affects migration decisions, but to a lesser extent, with t-values of 2.82 and 2.14, respectively.

Control variables provide additional insights. Age has an inverted U-shaped relationship with migration, suggesting that younger people are more likely to migrate while older people are less likely to migrate. Parental education is negatively related to migration, suggesting that those with more educated parents may have better opportunities locally, thus reducing the need to migrate. Political orientation, as measured by party membership, is associated with a lower probability of migration, which may be due to stronger social networks and local support for party members.

These results highlight the important role of individual and broader environmental health factors in influencing migration decisions. The consistency of these findings across models strengthens the relationship between health status and migrant preferences.

### Endogenous test

The potential endogeneity of health status in influencing migration preferences was addressed through various robustness checks. One concern is the potential bias in self-reported health measures. To mitigate this, we used an alternative health measure based on respondents’ experiences of physical pain affecting daily activities, as captured by the question: “In the past month, did physical pain problems affect your work or other daily activities?” This alternative measure, labeled Health_1, was used in the regression analysis, with results presented in Table 2. The findings remain robust, indicating that healthier individuals are more likely to migrate, and the endogeneity concerns do not significantly impact the results.

**Table 2:** Estimated results for endogenous tests.

Variables	OLS (1)	Probit (2)	Logit (3)	2SLS (4)	OLS (5)
Health				0.183***	0.00586*
				(4.32)	(1.75)
Health_1	0.00891***	0.0553***	0.112***		
	(3.17)	(2.90)	(3.12)		
Age	0.00475***	0.0515***	0.129***	0.00927***	0.00485***
	(4.50)	(5.89)	(7.18)	(5.90)	(4.59)
Age2	–	–	−0.00222***	–	–
	0.0000991***	0.000923***		0.000106***	0.0001000***
	(−7.81)	(−7.63)	(−8.62)	(−7.42)	(−7.88)
Politic	−0.0205**	−0.127*	−0.281**	−0.0399***	−0.0195**
	(−2.32)	(−1.92)	(−2.21)	(−3.58)	(−2.20)
Edu	0.00119	0.00322	−0.00607	−0.00292	0.00116
	(0.67)	(0.36)	(−0.37)	(−1.40)	(0.66)
Parents_edu	−0.00423**	−0.0258**	−0.0452**	−0.00487**	−0.00416**
	(−2.38)	(−2.31)	(−2.16)	(−2.43)	(−2.34)
Province_FE	Yes	Yes	Yes	Yes	Yes
_cons	0.156***	−1.419***	−2.961***	−0.661***	0.170***
	(4.44)	(−6.65)	(−7.37)	(−3.29)	(4.80)
*N*	11,635	11,635	11,635	11,634	11,635
Weak identification test (Cragg-Donald Wald F statistic)				46.609	–

Robust standard errors cluster at individual level. *t* statistics in parentheses. *p < 0.10, **p < 0.05, ***p < 0.01.

Moreover, to address potential reverse causality—whereby migration itself could improve health through better economic conditions and access to healthcare—we employed instrumental variables (IV) for health. Specifically, we used smoking and alcohol consumption habits as instruments, as these behaviors affect health but are not directly related to migration decisions. The results from the IV regression, also reported in Table 3, show that health continues to positively influence migration, with a significant increase in the probability of migration associated with better health.

**Table 3:** The moderating effect of class mobility expectations on migration preference.

	(7)	(8)	(9)	(10)	(11)	(12)
	Emotion	imm	Generation	imm	Generation	imm
Health	0.104***	0.00457	0.0219***	0.00706*	0.0121*	0.00543
	(8.58)	(1.27)	(4.01)	(2.01)	(2.42)	(1.54)
Sex	0.200***	0.0125*	−0.00526	0.00210	−0.0107	0.00251
	(10.47)	(2.03)	(−0.56)	(0.34)	(−1.29)	(0.41)
Politic	0.278***	−0.0221**	0.0173	−0.0200*	−0.0236	−0.0210*
	(8.01)	(−2.61)	(1.13)	(−2.29)	(−1.64)	(−2.42)
parents_edu	0.0269***	−0.00195	−0.0143***	−0.00509**	−0.0106***	−0.00331
	(4.45)	(−1.12)	(−5.02)	(−2.99)	(−4.04)	(−1.92)
Age	0.00311	0.00667***	−0.00281	0.00500***	−0.00246	0.00575***
	(0.78)	(6.08)	(−1.51)	(4.64)	(−1.49)	(5.34)
age2	0.000583 (1.14)	−0.0012*** (−8.99)	0.0000886 (0.39)	- 0.00103*** (−7.96)	−0.00062** (−2.98)	−0.000106** (−8.26)
f1friend		−0.0176***				
		(−6.07)				
hope1				−0.00356		
				(−1.56)		
hope2						0.0141***
						(5.95)
Province_F E	Yes	Yes	Yes	Yes	Yes	Yes
_cons	1.321***	0.174***	0.501***	0.171***	0.844***	0.146***
	(10.81)	(4.64)	(8.95)	(4.58)	(16.55)	(3.80)
*N*	10,210	10,210	11,635	10,679	11,635	10,591

Individual Robust standard errors in parentheses. *t* statistics in parentheses. *p < 0.10, **p < 0.05, ***p < 0.01.

**Figure 4:**
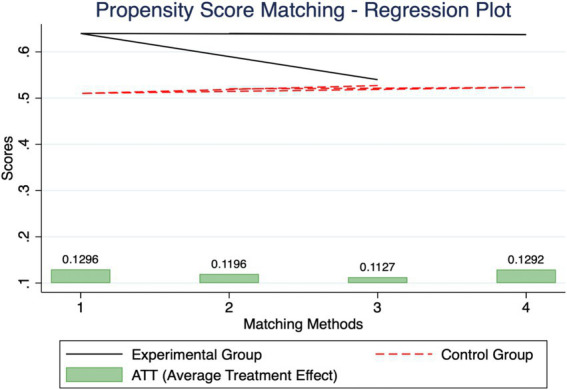
Propensity score matching.

To address potential self-selection bias, we estimated the average treatment effect (ATT) of health on migration using propensity score matching (PSM). The results, presented in Figure 4, show that individuals with better health are significantly more likely to migrate. Sensitivity analyses further reinforce the robustness of these findings, suggesting that unobserved confounders are unlikely to have biased the estimates Heckman (30).

The PSM analysis consistently demonstrates a significant positive treatment effect across all matching methods, with the ATT indicating an increase in the likelihood of migration by approximately 11 to 13%. These effects are statistically significant at the *p* < 0.01 level for Nearest Neighbor, Radius, Caliper, and Nuclear matching methods, underscoring the reliability of the treatment effect. The relatively small variation percentages suggest a stable impact, while Gamma values ranging from 2.9 to 3.2 indicate that the results are resilient to potential unobserved confounders.

Overall, the PSM analysis strongly supports the conclusion that better health positively influences migration outcomes.

### The impact of health status, and social capital on migration preference

The results demonstrate that health status significantly influences migration decisions, with social capital factors such as occupational quality, social networks, and sense of fairness serving as key mediators, and class mobility expectations acting as a moderator. As shown in Table 4, individuals with better health are more likely to migrate. Specifically, healthier individuals tend to enhance their occupational quality through vocational training, which in turn increases their likelihood of migration. For example, the regression analysis indicates that a one-unit increase in self-rated health is associated with a significant improvement in occupational quality (*β* = 0.0119, *p* < 0.01), which subsequently raises the probability of migration. This finding aligns with the hypothesis that individuals in better health are more inclined to seek and secure better employment opportunities, often necessitating relocation to areas with higher wages, thus validating Hypothesis 1.

**Table 4:** The mechanism of social capital on migration preference.

	(1)	(2)	(3)	(4)	(5)	(6)
	Occupation	imm	Social network	imm	Equality	imm
Health	0.0119**	0.00569	0.0727***	0.00330	0.141***	0.00670*
	(3.20)	(1.69)	(8.55)	(0.86)	(12.95)	(1.98)
Sex	0.0602***	0.000594	0.296***	0.000922	−0.0521**	0.00136
	(8.89)	(0.10)	(20.33)	(0.13)	(−2.99)	(0.23)
Politic	0.222***	−0.0215*	0.276***	−0.0299***	0.0330	−0.0174*
	(16.02)	(−2.47)	(11.89)	(−3.29)	(1.11)	(−2.06)
parents_ed u	0.0313*** (12.96)	−0.00426* (−2.53)	0.0682*** (15.10)	−0.00727*** (−3.82)	0.00633 (1.17)	−0.00367* (−2.21)
Age	0.0176*** (15.07)	0.00466*** (4.39)	0.00977** (3.11)	0.00228 (1.78)	−0.0356*** (−10.54)	0.00477*** (4.55)
age2	−0.00026*** (−18.75)	−0.000973*** (−7.61)	−0.00297*** (−8.02)	−0.000739*** (−4.94)	0.00458*** (10.85)	−0.00992*** (−7.89)
Train		0.0177* (2.03)				
f1soc				0.0158** (3.20)		
Fair						−0.00572 (−1.82)
Province_ FE	Yes	Yes	Yes	Yes	Yes	Yes
_cons	−0.189***	0.175***	0.762***	0.237***	3.148***	0.189***
	(−4.61)	(4.90)	(8.47)	(5.75)	(29.98)	(5.15)
*N*	11,635	11,635	9,289	9,289	11,635	11,635

Cluster individual robust standard errors in parentheses. *t* statistics in parentheses. *p < 0.10, **p < 0.05, ***p < 0.01.

Furthermore, health status positively impacts the breadth and strength of social networks, as indicated by the results in Table 4 (*β* = 0.0727, *p* < 0.001). Individuals with larger social networks are more likely to migrate, as these networks provide essential support, information, and resources that facilitate the migration process. This underscores the role of social capital in making migration a more feasible and attractive option for healthier individuals, supporting Hypothesis 2.

However, a sense of fairness also plays a critical role in migration decisions. The findings suggest that while better health enhances individuals’ perceptions of fairness in their current environment (*β* = 0.141, *p* < 0.001), this increased perception of fairness is associated with a lower likelihood of migration. This implies that those who perceive their current situation as fair and adequately compensated are less motivated to relocate, opting instead for the stability and security of their present environment.

### The moderating effect of class mobility expectations on migration preference

Table 3 further explores the moderating effect of class mobility expectations on the relationship between health and migration. The analysis shows that individuals who are in better health and expect to ascend the social class ladder are less likely to migrate (*β* = 0.0219, *p* < 0.001), as they anticipate better outcomes in their current position. Conversely, those who have already experienced an upward shift in class position are more likely to migrate (*β* = 0.0121, *p* < 0.05). This suggests that individuals who have already achieved some success feel more confident in their ability to further improve their circumstances, even if it requires relocation. Additionally, stronger emotional support networks, which are more prevalent among healthier individuals, appear to reduce the likelihood of migration (*β* = 0.104, *p* < 0.001), possibly due to the comfort and stability these networks provide.

Overall, the findings highlight the complex and multifaceted interactions between health, social capital, and class mobility expectations in shaping migration preferences. Healthier individuals are generally more likely to migrate, supported by improved occupational quality and broader social networks. However, factors such as an increased sense of fairness and strong emotional support networks can mitigate this effect by providing stability in the current environment. Class mobility expectations further moderate this relationship, with optimism about prospects discouraging migration and the realization of upward mobility encouraging it, which validates Hypotheses 2 and 3.

## Discussion

This study contributes to a deeper understanding of the complex relationship between health status, social capital, and migration preferences, a topic that has been underexplored in the literature. While the role of economic and geographic factors in migration decisions has been widely studied, this research expands the discussion by examining the mediating effects of occupational quality, social networks, and perceived fairness, as well as the moderating role of class mobility expectations.

Our findings confirm that individuals in better health are more likely to migrate, a trend that is strongly influenced by the quality of their employment opportunities. Healthier individuals tend to pursue vocational training or higher education, which in turn enhances their access to better job prospects in regions offering higher wages. This aligns with recent studies suggesting that health is intricately linked to life decisions, including migration, as it facilitates the pursuit of higher-quality employment opportunities (42, 43). These findings reinforce the notion that physical well-being acts as a driver for migration, particularly when individuals can capitalize on better work opportunities (20).

Another critical factor that emerged is the role of social networks. Our study finds that individuals in better health and with stronger social networks are more likely to migrate. Social networks provide vital support, information, and resources that help individuals overcome the challenges associated with relocation. This finding is consistent with recent studies on migration, which emphasize the importance of social capital, especially in facilitating migration decisions among younger adults and other highly mobile populations (21, 22). These networks provide a sense of security and reassurance, making the migration process more manageable and appealing.

However, an interesting and somewhat counterintuitive result was that a sense of fairness in one’s current environment significantly influences the decision to migrate. The study found that while better health increases perceptions of fairness, this enhanced sense of fairness was associated with a lower likelihood of migration. When individuals perceive their current situation as fair and equitable, they tend to be more satisfied with their circumstances and less inclined to seek opportunities elsewhere. This supports the argument that psychological and social factors, such as a sense of security and perceived fairness, can mitigate the desire for migration (23, 24). Individuals who feel their needs are met in their current environment often prefer stability over the potential risks of relocating.

An important contribution of this study is its exploration of how class mobility expectations shape the relationship between health and migration. We found that individuals in better health who expect to move up the social ladder in their current location tend to stay, as they believe they can achieve their goals without migrating. Conversely, those who have already experienced upward mobility are more likely to consider migration, as they have greater confidence in their ability to improve their situation through relocation. These findings align with recent literature on migration choices, which highlights the role of past achievements and future expectations in shaping individuals’ migration intentions (8, 25, 26). This suggests that migration decisions are not only shaped by immediate circumstances but also by the expectations and aspirations individuals hold for their future social mobility.

Moreover, the study also reveals that stronger emotional support networks, which are often more prevalent among healthier individuals, deter migration. These networks provide a sense of emotional security, which may reduce the perceived need for relocation. The importance of emotional support, particularly in times of transition, cannot be understated, as it offers individuals the confidence to navigate life challenges without needing to migrate (27).

## Limitations

Despite the significant contributions of this study, several limitations must be acknowledged. First, the study relies on self-reported health data and other variables, which introduces the potential for bias. Self-reports are inherently subjective and can be influenced by factors such as social desirability bias, respondents’ self-awareness, and personal interpretation of health-related questions. Individuals may underreport or overreport their health status, which can lead to inaccuracies in the data. While we attempted to mitigate this issue by using external assessments, the limitations of self-reported health remain. Future research could benefit from utilizing objective health measures, such as medical records or clinical assessments, which would help validate the self-reported data and provide a more accurate picture of participants’ health.

Second, the study did not account for physical activity levels, a crucial factor that could influence both health status and migration decisions. Physical activity has been shown to have a direct impact on both physical and mental health, and it likely plays a role in an individual’s decision to migrate, particularly when considering the physical demands of relocation. The absence of physical activity data in this study may limit our understanding of how it interacts with health and migration preferences. Therefore, future research should incorporate detailed assessments of physical activity, including frequency, intensity, and type, to better capture its potential influence on the health-migration relationship.

Third, the study is based on cross-sectional data, which limits the ability to establish causality. The cross-sectional nature of the data provides a snapshot of the relationship between health and migration preferences at a single point in time, but it cannot address the directionality of the relationship. It is possible that migration decisions affect health, rather than the other way around. To address this limitation, longitudinal studies are needed to capture changes in migration preferences over time, enabling a more dynamic understanding of how health status, social capital, and migration decisions interact. Longitudinal designs would allow for the exploration of causality and the identification of temporal patterns in the health-migration relationship.

Finally, the scope of social media usage was limited to the devices most frequently used by participants, potentially excluding other forms of social media engagement. Social media usage, particularly through mobile devices and diverse platforms, plays an increasingly important role in shaping individuals’ perceptions and decisions, including migration decisions. By restricting the scope of social media assessment to only the primary devices used by participants, the study may have overlooked important aspects of how social media influences migration preferences. Future research should consider a more comprehensive assessment of social media usage across various devices and platforms, including emerging platforms and mobile applications, to provide a fuller picture of how social media affects migration decisions in today’s digital landscape.

## Conclusion

This study provides a comprehensive analysis of the impact of health status on immigration preferences using a multiple logit regression model. The findings consistently show that individual health levels significantly and positively influence the likelihood of choosing to migrate. Individuals in better health are more inclined to pursue mobility, and this relationship remains robust even after addressing potential endogeneity and self-selection bias through instrumental variable approaches and propensity score matching.

Interestingly, the study also uncovers that parental education levels negatively influence mobility behavior. In cases where parents can offer better employment opportunities locally, individuals are less likely to leave their hometowns, particularly under the traditional mindset of “settling down and moving to a new place.” This highlights the role of familial and social factors in shaping migration decisions. Further analysis reveals that health impacts migration preferences through various mechanisms, including occupational quality, sense of fairness, social networks, emotional support networks, and expectations of class mobility. The effect of health on migration also exhibits heterogeneity based on factors such as skill level, education, family capital, and age. Specifically, younger and middle-aged individuals are more likely to migrate, often seeking better opportunities in more developed regions, which are perceived as offering higher salaries and living standards. Health, therefore, plays a crucial role in determining an individual’s ability to pursue these opportunities and achieve a better quality of life.

In contrast to much of the existing literature, which has often overlooked the significant influence of health status on migration preferences, this study integrates the Harris-Todaro model to develop a migration preference function that highlights the relationship between health and migration. The implications of these findings are multi-faceted. First, migration is a key factor in the economic development of countries, and ensuring robust health and social security systems is essential for facilitating migration and supporting economic growth. Enhancing health security among migrant populations is crucial to ensuring their well-being and capacity to contribute economically. Additionally, there is a need to integrate various levels of medical insurance to create a more cohesive health support system for urban workers, urban residents, and rural populations. Based on the social network mechanisms identified in this study, governments should establish strong social support networks for migrants, encourage their social participation, and address their mental health and emotional needs. This can help mitigate challenges such as job instability and lack of social support, which are detrimental to both the physical and mental health of migrant populations. Finally, addressing health inequalities between migrants and resident citizens is vital for fostering inclusive economic development, particularly in developing countries.

This study’s focus on China presents some limitations, as the findings may not be generalizable to other contexts. Future research should explore the relationship between health status and migration preferences in other regions, with particular attention to health education inequalities and broader health literacy.

## Data Availability

The dataset presented in this study is a public dataset. This data can be found here: http://css.sysu.edu.cn. If needed the data can also be requested by sending an official email to : cssdata@mail.sysu.edu.cn.

## Footnotes

^1^China immigration means internal immigration from rural to urban.

^2^Identification policy across China means Household registration policy generates social security costs.
